# Contribution of CARD9 signaling to wound healing in skin promoted by topical administration of heat-killed *Enterococcus faecalis* strain KH2 and the involvement of Dectin-2

**DOI:** 10.3389/fimmu.2025.1550934

**Published:** 2025-06-12

**Authors:** Shiho Kurosaka, Hiromasa Tanno, Minako Hirose, Wakana Kamada, Rena Takayashiki, Ikue Sone, Yuki Sato, Takumi Watanabe, Shinyo Ishi, Miki Shoji, Yoshimichi Imai, Ko Sato, Keiko Ishii, Hiromitsu Hara, Sho Yamasaki, Shinobu Saijo, Yoichiro Iwakura, Kazuyoshi Kawakami, Emi Kanno

**Affiliations:** ^1^ Department of Translational Science for Nursing, Tohoku University Graduate School of Medicine, Sendai, Japan; ^2^ Bio-Lab Co., Ltd., Hidaka, Japan; ^3^ Department of Plastic and Reconstructive Surgery, Tohoku University Graduate School of Medicine, Sendai, Japan; ^4^ Department of Medical Microbiology, Mycology and Immunology, Tohoku University Graduate School of Medicine, Sendai, Japan; ^5^ Department of Immunology, Graduate School of Medical and Dental Sciences, Kagoshima University, Kagoshima, Japan; ^6^ Department of Molecular Immunology, Research Institute for Microbial Diseases, Osaka University, Suita, Japan; ^7^ Laboratory of Molecular Immunology, Immunology Frontier Research Center, Osaka University, Suita, Japan; ^8^ Division of Molecular Immunology, Medical Mycology Research Center, Chiba University, Chiba, Japan; ^9^ Department of Veterinary Medical Sciences, Graduate School of Agricultural and Life Sciences, The University of Tokyo, Tokyo, Japan; ^10^ Laboratory for Human Disease Models, Louis Pasteur Center for Medical Research, Kyoto, Japan

**Keywords:** skin wound healing, *Enterococcus faecalis* KH2, C-type lectin receptors, CARD9, Dectin-2

## Abstract

**Introduction:**

Lactic acid bacteria (LAB) are well known for their beneficial effects on the regulation of immune responses and host protection against microbial infections. We previously reported that heat-killed *Enterococcus faecalis* strain KH2 (heat-killed KH2), a species of LAB, enhances inflammatory responses at wound sites and accelerates the skin wound healing process. In this study, we aimed to clarify the pathway underlying the wound-healing effects of heat-killed KH2. We focused on CARD9, a common adaptor molecule for C-type lectin receptors and Dectin-2, the upstream receptor for this adaptor molecule.

**Methods:**

Four full-thickness dermal wounds were created on the backs of wild-type (WT) mice, CARD9 KO mice, and Dectin-2 KO mice, and the effects of heat-killed KH2 administration were examined. We analyzed the percent wound closure, re-epithelialization, granulation tissue formation, and the production of inflammatory cytokines and chemokines.

**Results:**

Heat-killed KH2 administration enhanced wound closure, granulation tissue formation, and re-epithelialization in WT mice. However, these effects were absent in heat-killed KH2-treated CARD9 KO mice. Similar results were observed in the migration of neutrophils and the production of TNF-α, IL-6, KC, and MIP-2 in heat-killed KH2-treated CARD9 KO mice. Furthermore, heat-killed KH-2 induced activation of reporter cells expressing Dectin-2. Finally, heat-killed KH-2 treatment in Dectin-2 KO mice did not promote skin wound healing.

**Conclusion:**

These results suggest that recognition of heat-killed KH2 by Dectin-2 may activate CARD9-mediated signaling, which may contribute to the promotion of skin wound healing through KH2 treatment.

## 1 Introduction

Skin wound healing consists of three phases: inflammation, proliferation, and remodeling (1). In the inflammatory phase, keratinocytes and fibroblasts produce inflammatory cytokines and chemokines, such as tumor necrosis factor (TNF)-α, interleukin (IL)-6, keratinocyte-derived chemokine (KC), and macrophage inflammatory proteins (MIP)-1 and MIP-2. These molecules are important for leukocyte recruitment, and the migrated leukocytes, including neutrophils and macrophages, phagocytose pathogens and necrotic tissue, help to cleanse the wound and protect against infection. In the proliferative phase, numerous growth factors are essential for the formation of granulation tissue, angiogenesis, and epithelialization, which play critical roles in the healing process (1, 2). Lactic acid bacteria (LAB) are well known for their beneficial effects on intestinal regulation, anti-allergic activity, host protection against microbial infections, and anti-tumor effects (3, 4). LAB are generally classified into several groups, including *Lactobacillus* spp., *Lactococcus* spp., *Streptococcus* sp., and *Enterococcus* spp (5). Several studies have demonstrated the beneficial effects of LAB on wound healing. For example, genetically modified *Lactobacillus* enhances wound repair in a mouse and pig wound model (6, 7). Topical administration of heat-killed *L. plantarum* accelerates skin wound healing by increasing macrophage infiltration at the wound sites (8). We previously examined the effect of heat-killed *E. faecalis* strain KH2 (heat-killed KH2) on skin wound healing and reported that heat-killed KH2 induces inflammatory cytokines including TNF-α and IL-6 and promotes skin wound healing by accelerating re-epithelialization and granulation tissue formation (9). However, the mechanism by which heat-killed KH2 induces inflammation and promotes wound healing is not yet fully understood. In general, LABs are recognized by pattern recognition receptors, such as Toll-like receptors (TLRs) and C-type lection receptors (CLRs). TLRs are the most commonly reported pattern recognition receptors that recognize LAB (10, 11). However, in recent years, several studies have demonstrated the involvement of CLRs in the recognition of LAB (12, 13). There are various types of CLRs, including dendritic cell-associated C-type lectin (Dectin)-1, Dectin-2, Dectin-3, and macrophage-inducible C-type lectin (Mincle). Dectin-1, 2, 3, and Mincle are mainly expressed on dendritic cells (DCs) and macrophages. When ligands bind to these CLRs, these cells produce inflammatory cytokines via caspase recruitment domain-containing protein 9 (CARD9), a downstream signaling adaptor molecule of CLRs (14). We have previously shown that the CLRs-CARD9 pathway is involved in the skin wound healing process (15, 16).

Based on this background, in this study, we aimed to define the roles of CLRs and CARD9 in wound healing. We found that heat-killed KH2 is recognized by Dectin-2, not Dectin-1, and that CARD9-mediated signaling is essential for the promotion of skin wound healing by heat-killed KH2.

## 2 Materials and methods

### 2.1 Animals

CARD9, Dectin-1, and Dectin-2 KO mice were generated as previously described (17, 18). These KO mice were backcrossed to C57BL/6 mice for more than eight generations. Wild-type (WT) C57BL/6 mice, obtained from CLEA Japan (Tokyo, Japan), were used as controls. Male and female mice, aged 7 to 10 weeks, were housed under specific pathogen-free conditions at the Institute for Animal Experimentation, Tohoku University Graduate School of Medicine (Sendai, Japan) with *ad libitum* access to food and water. All animal experiments were approved by the Ethics Review Committee for Animal Experimentation of Tohoku University and were performed under anesthesia to minimize animal suffering.

### 2.2 Preparation of heat-killed *E. faecalis* KH2 and dextrin

*E. faecalis* KH2 (NITEP-14444; GenBank AB534553), isolated from a human fecal sample, was obtained from Bio-Lab Co., Ltd. (Saitama, Japan) (19). The strain was cultured aerobically overnight at 37°C in MRS broth (Difco, Detroit, MI, USA), centrifuged, and heat-killed at 105°C for 30 min using an autoclave (HV-2IILB; Hirayama Manufacturing Corp., Saitama, Japan). To improve water dispersibility, the heat-killed bacteria were homogenized at 15 MPa (ECONIZER LABO-01; Sanmaru Machinery Co., Ltd., Shizuoka, Japan) and mixed with an equal amount of dextrin (NSD300; San-ei Sucrochemical Co., Ltd., Aichi, Japan). The final suspension was diluted to 200 mg/mL with normal saline.

### 2.3 Wound creation and tissue collection

As previously described (9), mice were anesthetized with isoflurane (1.0–2.0%; Mairan Pharma, Osaka, Japan) and the dorsal skin was shaved and sterilized with 70% ethanol. Four 6-mm full-thickness wounds were created using a biopsy punch (Kai Industries Co., Ltd., Gifu, Japan). The wounds were covered with a polyurethane film (Tegaderm Transparent Dressing, 3M, Health Care, Saint Paul, MN, USA) for occlusive dressing, and an elastic bandage (Hilate, Iwatsuki, Tokyo, Japan) was applied to prevent the film from peeling off. At specified time points, mice were euthanized, and 1 cm² skin samples were excised for histopathological analysis and cytokine quantification.

### 2.4 Heat-killed KH2 treatment of wounds

Wounds were created following the method described above. Immediately after wounding, a 5-μL suspension of heat-killed KH2 (1000 μg), or an equal volume of dextrin as a vehicle control, was applied to the base of the wound in mice using a pipette.

### 2.5 Measurement of the wound area

Digital images of the wounds were acquired at baseline and at designated time points using a digital camera (CX4, Ricoh, Tokyo, Japan). The wound area was quantified using ImageJ software (NIH, Bethesda, MD, USA). Wound healing was evaluated as percent wound closure, calculated as follows: % wound closure = (1 − wound area at the indicated time point/wound area on day 0) × 100.

### 2.6 Histopathology and immunohistochemistry

The wounded tissues were fixed in 4% paraformaldehyde, paraffin-embedded, sectioned, and stained with hematoxylin-eosin (HE) (16, 20). Re-epithelialization was assessed by measuring the distance from the wound edge to the advancing epithelial cells in HE-stained sections. The re-epithelialization index was calculated as the percentage of the wound area covered by new epithelium. The granulation tissue area was also quantified from the HE-stained sections.

### 2.7 Measurement of cytokine and chemokine concentrations

The wound tissues were homogenized with a saline solution using a stainless-steel mesh, and the supernatant was collected after centrifugation. Supernatants were analyzed for cytokine and chemokine levels using enzyme-linked immunosorbent assay (ELISA) kits (BioLegend, San Diego, CA, USA, for TNF-α and IL-6; R&D Systems, Minneapolis, MN, USA, for KC, MIP-2, MIP-1α, and MIP-β). The results were expressed as values per wound.

### 2.8 Preparation of leukocytes in the wound tissue

As previously described (8), mice were sacrificed on days 1, 3 and 7 post-wounding. Wound tissues were excised, minced, and digested with a mixture of enzymes (Roche, Mannheim, Germany, 0.2 mg/mL Liberase TL, 2.5 mg/mL collagenase D, 0.1 mg/mL DNase I, 2.0 mg/mL Dispase II) in RPMI 1640 medium (Sigma-Aldrich, St. Louis, MO, USA) supplemented with 10 mM HEPES (Sigma) and 10% fetal calf serum (FCS; Biowest, Nuaillé, France). Cell suspensions were filtered through a 70 μm cell strainer (BD Falcon, Bedford, MA, USA), washed, and used for flow cytometric analysis.

### 2.9 Flow cytometric analysis

The cells obtained from the wounds were incubated with anti-mouse CD16/CD32 (clone 2.4G2, BD Biosciences, Franklin Lakes, NJ, USA) on ice for 15 min in phosphate-buffered saline (PBS) that contained 1% FCS and 0.1% sodium azide. The cells were then stained with the following antibodies: APC/Cy7-anti-Ly6G mAb (clone 1A8, BioLegend), APC-anti-CD11b mAb (clone M1/70, BioLegend), PE-anti-F4/80 mAb (clone BM8, BioLegend), Pacific Blue-anti-CD45 mAb (clone 30-F11, BioLegend), FITC-anti-CD45R/B220 mAb (clone RA3-6B2, BioLegend), FITC-anti-NK1.1 mAb (clone PK136, BioLegend), FITC-anti-T-cell receptor γδ (TCRγδ) mAB (clone GL3, BioLegend), FITC-anti-CD3ϵ mAb (clone 145-2C11, BioLegend), 7-AAD Viability Staining Solution (BioLegend), APC-anti-Ly6C mAb (clone HK1.4, BioLegend), and FITC- I-A/I-E (MHC II) (clone M5/114.15.2, BioLegend). To analyze macrophage subtypes, the CD45+ cells were gated on Ly6G-F4/80+ cells and subsequently analyzed the expression of Ly6C and MHC II within the Ly6G-F4/80+ population. Isotype-matched irrelevant IgG was used as a control for staining. The gating strategy is shown in **Supplementary Figures 1** and **2**.

### 2.10 Generation and culture of bone marrow-derived dendritic cells

BM-DCs were prepared following the protocol of Luts et al. (21). Briefly, BM cells from WT, CARD9 KO, Dectin-1 KO, and Dectin-2 KO mice were cultured at 2 × 10^5^ cells/mL in RPMI 1640 medium supplemented with 10% FCS, 100 U/mL penicillin G, 100 μg/mL streptomycin, and 50 μM 2-mercaptoethanol (Sigma-Aldrich) and 20 ng/mL murine granulocyte-macrophage colony-stimulating factor (GM-CSF; FUJIFILM Wako Pure Chemical Corporation, Osaka, Japan). On days 3 and 6, the cultures were supplemented with fresh medium containing GM-CSF. On day 8, non-adherent cells were harvested and used as BM-DCs. The obtained cells were then cultured with heat-killed KH2, LPS (Sigma-Aldrich), CpG1826 ODN (CpG) (Hokkaido System Science, Hokkaido, Japan), and heat-killed *Candida albicans* (HKCA) for 24 h at 37°C in a 5% CO_2_ incubator. The culture supernatants and cell pellets were collected and stored at -30°C before use.

### 2.11 Reporter assay

As described in previous reports (22), 2B4 T-cell hybridomas were transfected with an NFAT-GFP reporter construct, which consisted of three tandem NFAT-binding sites fused to enhanced GFP cDNA (23). This cell line was further transfected with Dectin-1 or Dectin-2 and FcRγ genes, and a control cell line lacking Dectin-2 was used. The cells were stimulated for 20 h in 37°C at 1.0 × 10^5^ cells/mL with heat-killed KH2 (1, 10, 100 μg/mL), zymosan-depleted (dZymosan; 60 μg/mL, InvivoGen, San Diego, USA), and furfurman (100 μg/mL, InvivoGen). The reporter cells were incubated with anti-mouse CD16/CD32 (clone 2.4G2, BD Biosciences) on ice for 15 min in PBS that contained 1% FCS and 0.1% sodium azide. The cells were then stained with APC-anti-CD3ϵ mAb (clone 145-2C11, BioLegend) and washed three times in the same buffer. Isotype-matched IgG was used for control staining. GFP expression in gated CD3-positive reporter cells was evaluated using a BD FACS Canto II flow cytometer (BD Bioscience).

### 2.12 Statistical analysis

Data were analyzed using JMP^®^ Pro 17 0.0 software (SAS Institute Japan, Tokyo, Japan). Results are expressed as the mean ± SD. Differences between groups were assessed for statistical significance using Dunnett’s or Tukey–Kramer’s honestly significant difference (HSD) test for comparisons involving more than three experimental groups after confirming normality using the Shapiro–Wilk test. A *p*-value of less than 0.05 was considered statistically significant.

## 3 Results

### 3.1 Effects of CARD9 deficiency on the promotion of skin wound healing by heat-killed KH2 administration

**Figure 1:**
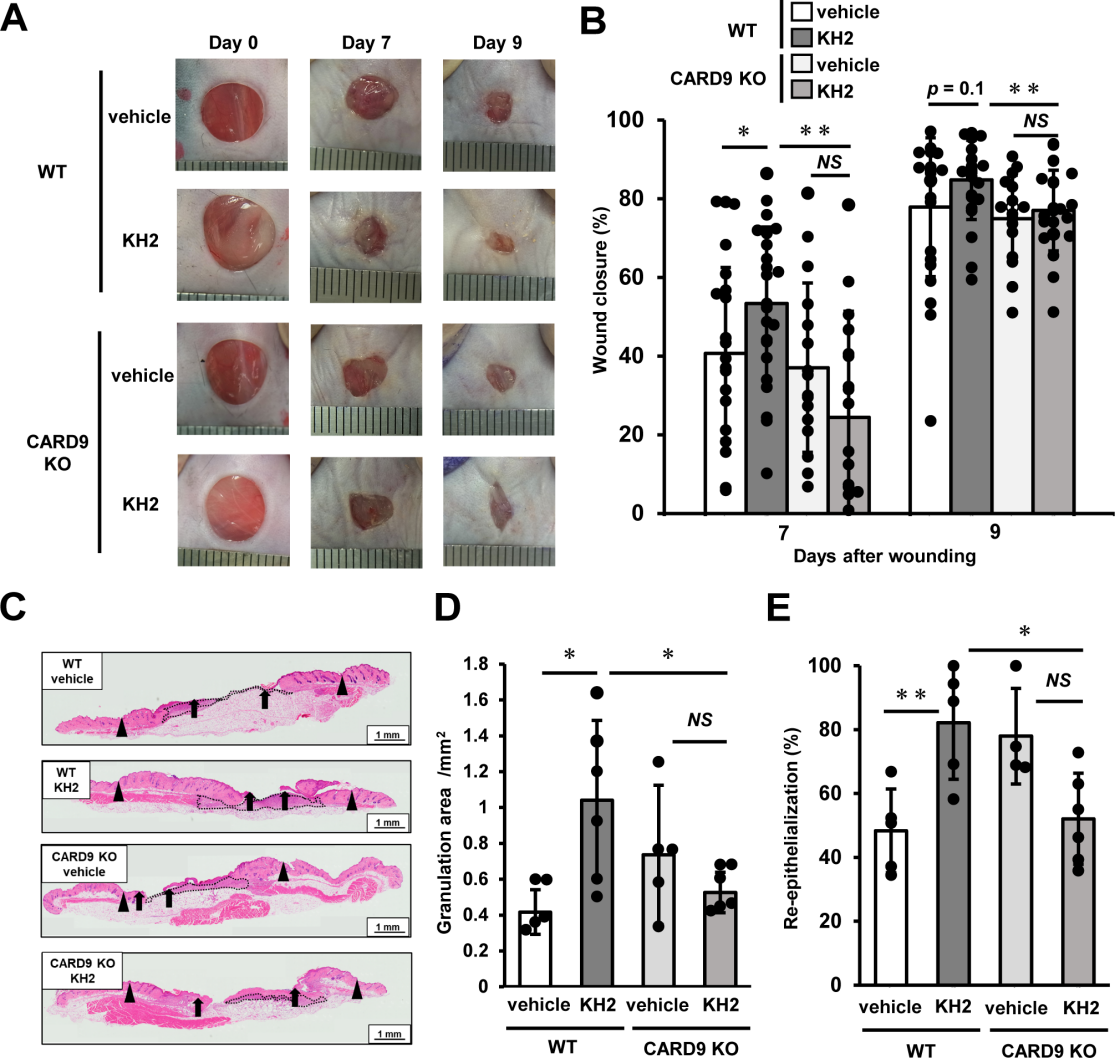
Effects of CARD9 deficiency on the promotion of skin wound healing by heat-killed KH2 administration. Wounds were created on the backs of WT mice or CARD9 KO mice treated with vehicle control or heat-killed KH2. Photographs **(A)** were taken, and the percentages of wound closure **(B)** were evaluated on days 7 and 9 after wound creation (n = 20–24 wounds). **(C)** Representative histological images of skin wounds on day 7. Arrowheads, arrows, and the dotted line indicate the original wound edges, granulation area, and re-epithelialized leading edges, respectively. The wound edges were defined as the border between normal epithelium and thick proliferative epithelium. **(D)** Granulation tissue area was quantified 7 days after wound creation (n = 4–6 wounds). **(E)** The re-epithelialization ratio was calculated 7 days post-wounding (n = 4–6 wounds). Each column represents the mean ± standard deviation. Results are representative of at least two independent experiments. *p < 0.05, **p < 0.01, NS, Not significant.

To investigate the involvement of CLRs in the recognition of heat-killed KH2 and the induction of inflammatory cytokines, we examined the role of CARD9, a downstream adaptor molecule of CLRs, by stimulating BM-DCs from WT and CARD9 KO mice with heat-killed KH2 and comparing TNF-α production. TNF-α production by BM-DCs upon stimulation with heat-killed KH2 was significantly reduced in CARD9 KO mice compared to WT mice (**Supplementary Figure 3**). Based on this result, we hypothesized that CLRs-CARD9 pathway is involved in the recognition of heat-killed KH2. Consequently, we considered the possibility that the CLRs-CARD9 pathway contributes to the mechanism by which KH2 promotes skin wound healing. To investigate this further, we used CARD9 KO mice to analyze the impact of CARD9 deficiency on the promotion of wound healing by heat-killed KH2. As shown in **Figures 1A, B**, WT mice treated with heat-killed KH2 exhibited significantly accelerated wound closure by day 7 post-wounding compared with WT mice treated with vehicle control. However, this effect was not observed in heat-killed KH2-treated CARD9 KO mice. In heat-killed KH2-treated WT mice, the granulation area and re-epithelialization rate were significantly increased by day 7 after wounding, compared with vehicle-treated WT mice. However, this effect was not observed in heat-killed KH2-treated CARD9 KO mice (**Figures 1C–E**). Additionally, wound closure, granulation area, and re-epithelialization rate were significantly delayed in heat-killed KH2-treated CARD9 KO mice compared to heat-killed KH2-treated WT mice (**Figures 1C–E**). These results suggest that CARD9 may be involved in the heat-killed KH2-mediated promotion of wound healing.

### 3.2 Effects of CARD9 deficiency on leukocyte infiltration by heat-killed KH2 administration

**Figure 2:**
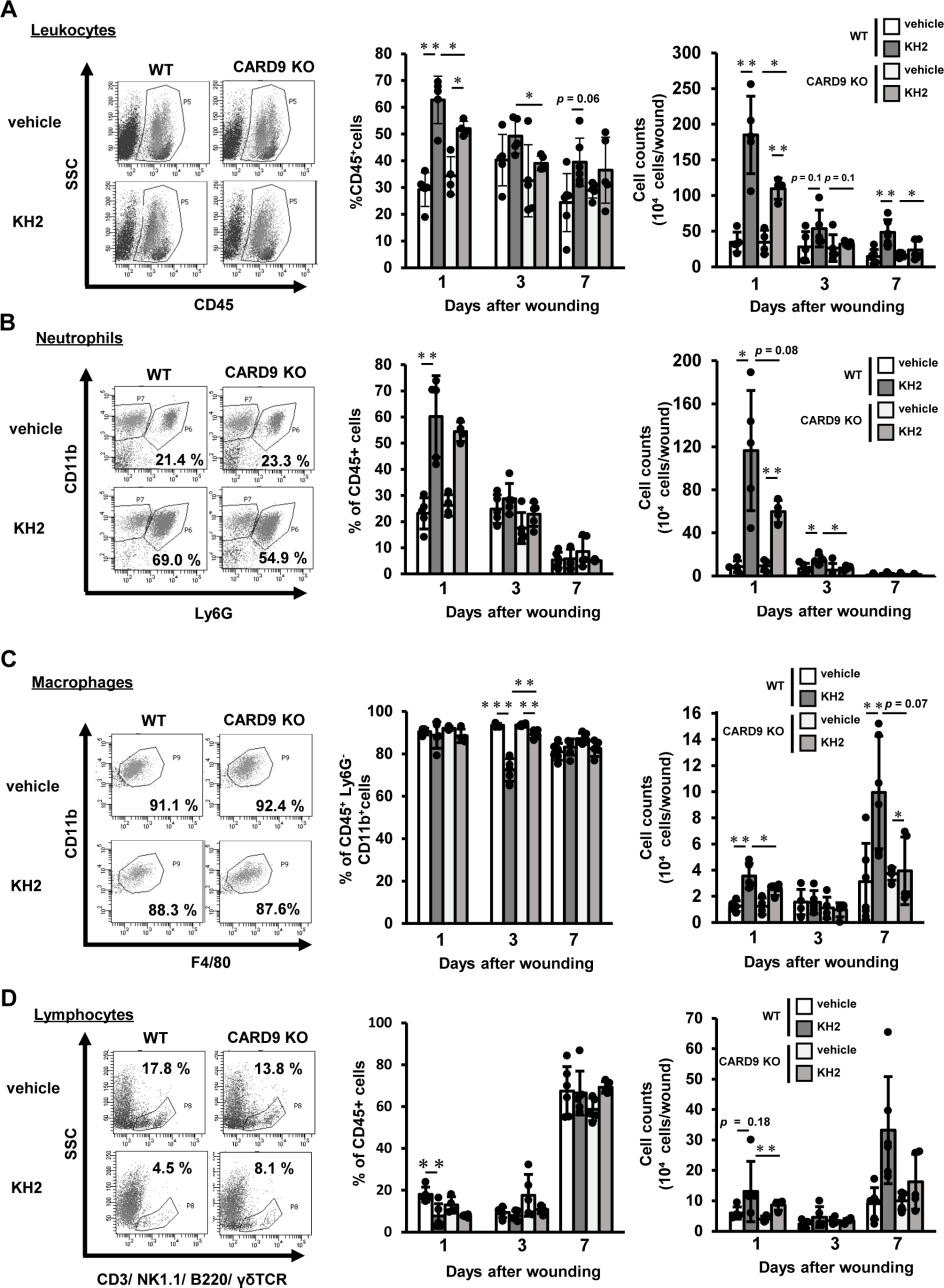
Effects of CARD9 deficiency on neutrophil infiltration by heat-killed KH2 administration. Flow cytometry was used to quantify the percentage and number of **(A)** leukocytes, **(B)** neutrophils, **(C)** macrophages, and **(D)** lymphocytes in wounded tissues on days 1, 3, and 7 post-wounding, as represented in the plots of wound-infiltrating cells. Leukocytes were prepared from four wounds at the indicated time points, and neutrophils were defined as CD45+Ly6G+CD11b+ cells. Macrophages were identified as CD45+F4/80+CD11b+Ly6G− cells. Lymphocytes were identified as CD45+ cells expressing CD3, NK1.1, TCRγδ, or B220 (n = 4–6 mice). The flow cytometry plots shown are representative of those from 1 day post-wounding. Each column represents the mean ± standard deviation. Results are representative of at least two independent experiments. *p < 0.05, **p < 0.01.

To elucidate the effects of CARD9 on heat-killed KH2-mediated leukocyte infiltration, we examined the kinetics of leukocyte infiltration in the wounded tissues. As shown in **Figure 2A**, the percentage of CD45+ leukocytes was significantly decreased in heat-killed KH2-treated CARD9 KO mice compared with heat-killed KH2-treated WT mice at 1 and 3 days post-wounding. The total number of leukocytes in the wounded tissues of heat-killed KH2-treated CARD9 KO mice was significantly reduced on day 1 post-wounding compared to heat-killed KH2-treated WT mice. There was no significant difference in the percentage of neutrophils between groups of heat-killed KH2-treated WT mice and CARD9 KO mice. On day 1, the number of neutrophils in heat-killed KH2-treated CARD9 KO mice showed a decreasing trend compared to heat-killed KH2-treated WT mice, with a significant reduction observed on day 3 (**Figure 2B**). The percentage of macrophages was significantly increased in heat-killed KH2-treated CARD9 KO mice compared with heat-killed KH2-treated WT mice at day 3. Additionally, the number of macrophages was significantly decreased in heat-killed KH2-treated CARD9 KO mice compared with heat-killed KH2-treated WT mice at day 1 post-wounding, with a trend toward a decrease at day 7 (**Figure 2C**). In contrast, there were no significant differences in the number of lymphocytes between heat-killed KH2-treated CARD9 KO and WT mice (**Figure 2D**). These results suggested that CARD9 may contribute to neutrophil accumulation in the early phase and macrophage accumulation in the late phase of wound healing after treatment with heat-killed KH2.

### 3.3 Effects of CARD9 deficiency on macrophage phenotype by heat-killed KH2 administration

**Figure 3:**
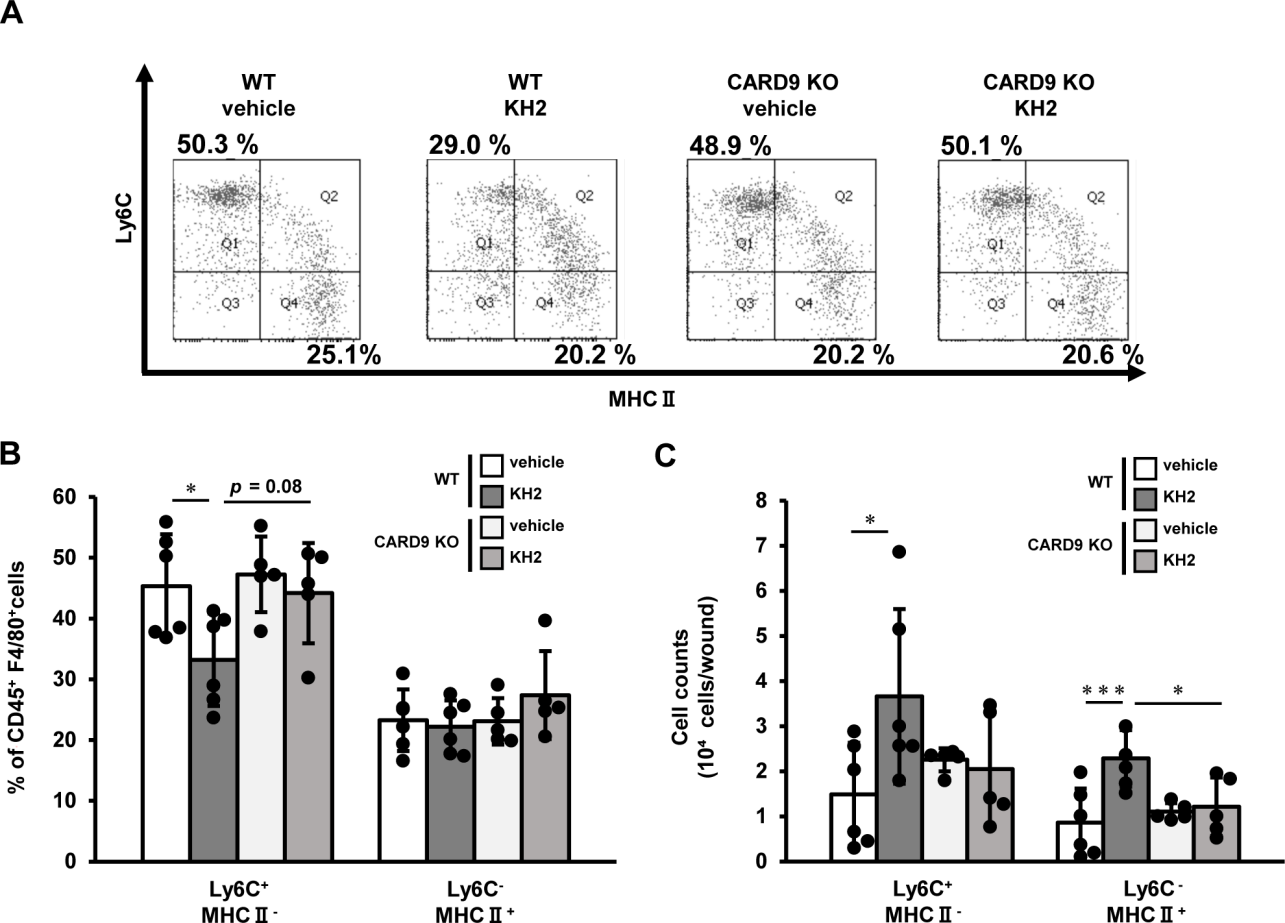
Effects of CARD9 deficiency on macrophage phenotype by heat-killed KH2 administration. Flow cytometry was used to quantify the percentage and number of macrophage subtypes in wounded tissues on day 7 after wound creation. **(A)** Representative plots of inflammatory and anti-inflammatory macrophages are shown. **(B)** The percentages of macrophages are shown, and **(C)** the number of macrophages is shown. Inflammatory macrophages were identified as CD45+F4/80+Ly6G−Ly6C+MHCII− cells. Anti-inflammatory macrophages were identified as CD45+F4/80+Ly6G−Ly6C−MHCII+ cells. (n = 5–6 mice). Each column represents the mean ± standard deviation. Results are representative of at least two independent experiments. *p < 0.05, ***p < 0.001.

Next, we analyzed macrophage subtypes at 7 days post-wounding. Following the classification by Rodero et al. (24), who reported Ly6C+MHCII− macrophages as the pro-inflammatory M1 phenotype and Ly6C−MHCII+ macrophages as the anti-inflammatory M2 phenotype, we also categorized macrophages accordingly. As shown in **Figures 3A–C**, the proportion of Ly6C+MHCII− macrophages was significantly lower in heat-killed KH2-treated WT mice compared with vehicle-treated WT mice, and also showed a trend toward a lower proportion compared with heat-killed KH2-treated KO mice. Furthermore, the numbers of Ly6C+MHCII− macrophages and Ly6C−MHCII+ macrophages significantly increased in heat-killed KH2-treated WT mice compared with vehicle-treated WT mice. However, this effect was not observed in heat-killed KH2-treated CARD9 KO mice. These results suggested that CARD9 may be crucial for heat-killed KH2 to modulate macrophage recruitment and polarization.

### 3.4 Effects of CARD9 deficiency on cytokine and chemokine production by heat-killed KH2 administration

**Figure 4:**
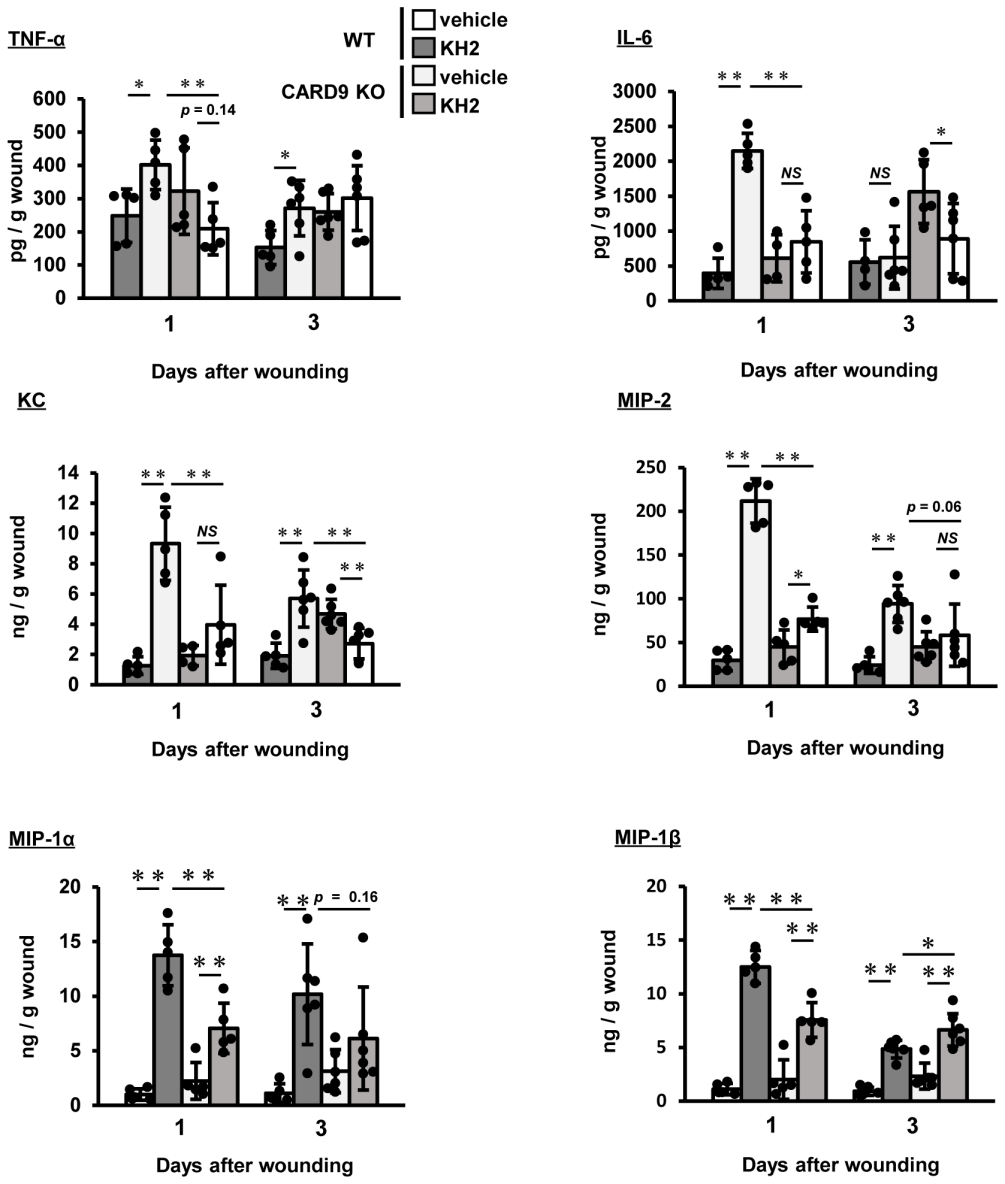
Effects of CARD9 deficiency on cytokine and chemokine production by heat-killed KH2 administration. Effects of CARD9 deficiency on the production of TNF-α, IL-6, KC, MIP-2, MIP-1α, and MIP-1β. Chemokine production in wound tissue homogenates was measured on days 1 and 3 post-wounding (n = 4–6 mice). Each column represents the mean ± standard deviation. Results are representative of at least two independent experiments. *p < 0.05, **p < 0.01, NS, Not significant.

Next, we evaluated how heat-killed KH2 treatment affected the synthesis of inflammatory cytokines and chemokines in the wounded tissues. As shown in **Figure 4**, TNF-α levels were significantly lower in heat-killed KH2-treated CARD9 KO mice compared to heat-killed KH2-treated WT mice on day 1 post-wounding. As shown in **Figure 4**, IL-6 was also significantly decreased in heat-killed KH2-treated CARD9 KO mice compared to heat-killed KH2-treated WT mice on days 1 and 3 after wounding. The production of KC and MIP-2, chemokines involved in neutrophil migration (25), was significantly reduced in heat-killed KH2-treated CARD9 KO mice compared to heat-killed KH2-treated WT mice on days 1 and 3 after wounding (**Figure 4**). The production of MIP-1α and MIP-1β, chemokines involved in macrophage migration (26), was significantly decreased in heat-killed KH2-treated CARD9 KO mice compared to heat-killed KH2-treated WT mice on day 1 post-wounding (**Figure 4**). These results indicated that CARD9 plays a key role in the early inflammatory response to heat-killed KH2, facilitating immune cell recruitment for wound healing.

### 3.5 Involvement of Dectin-1 and Dectin-2 in heat-killed KH2 recognition

To determine whether the loss of heat-killed KH2-mediated wound healing acceleration in CARD9 KO mice was due to the recognition of heat-killed KH2 by upstream CLRs, we examined the ability of Dectin-1 and Dectin-2 to recognize heat-killed KH2 using BM-DCs and an NFAT-GFP reporter assay.

**Figure 5:**
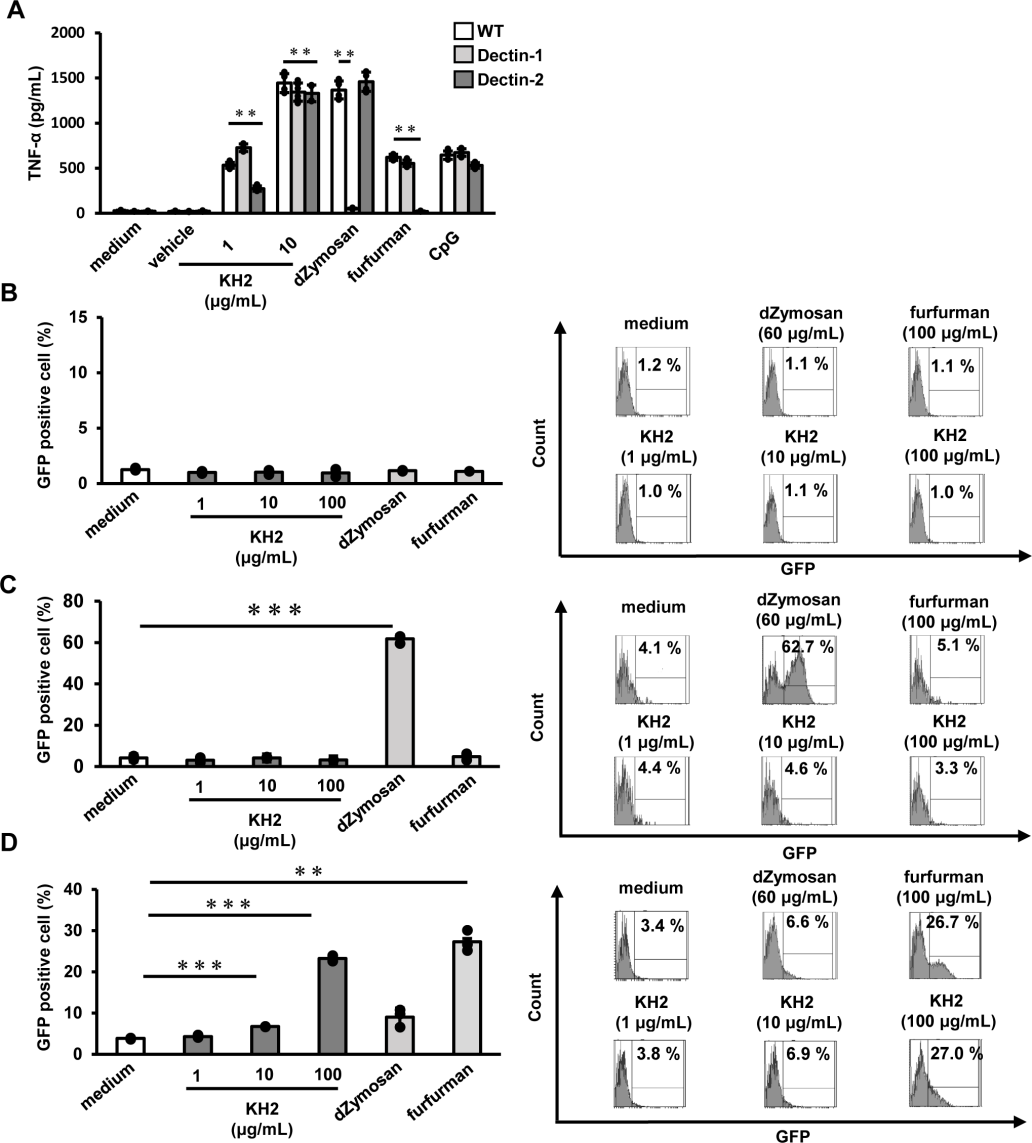
The effect of Dectin-1 and Dectin-2 deficiency on TNF-α production by heat-killed KH2 treatment by BM-DCs. Investigating the role of Dectin-1 and Dectin-2 reporter cells in response to heat-killed KH2. **(A)** BM-DCs were prepared from WT, Dectin-1 KO, and Dectin-2 KO mice and stimulated with heat-killed KH2 (1, 10 μg/mL), CpG (1 μg/mL), dZymosan (60 μg/mL) and furfurman (100 μg/mL) for 24 h. Production of TNF-α in the culture supernatants was analyzed. **(B)** Control, **(C)** Dectin-1, or **(D)** Dectin-2-NFAT-GFP reporter cells were cultured with heat-killed KH2, vehicle, dZymosan (60 μg/mL) and furfurman (100 μg/mL). GFP expression was measured by flow cytometry. dZymosan, which is a Dectin-1 ligand, furfurman, which is a Dectin-2 ligand, and CpG, which is a TLR9 ligand, were used as the positive controls. Representative histograms from three independent experiments are shown. Each column represents the mean ± standard deviation of triplicate cultures. Results are representative of at least two independent experiments. **p < 0.01, ***p < 0.001.

Stimulation of BM-DCs from Dectin-1 or Dectin-2 KO mice with heat-killed KH2 for 24 h revealed TNF-α production, as measured by ELISA. TNF-α production in heat-killed KH2 stimulated BM-DCs from Dectin-1 KO mice was comparable to that of BM-DCs from WT mice. While the positive control dZymosan (Dectin-1 ligand) stimulation resulted in decreased TNF-α production, stimulation with the non-Dectin-1 ligands furfurman (Dectin-2 ligand) and CpG (TLR9 ligand) resulted in TNF-α production in Dectin-1 KO mice comparable with that observed in WT mice. On the other hand, heat-killed KH2-induced TNF-α production was significantly reduced in BM-DCs from Dectin-2 KO mice. Furthermore, stimulation with the Dectin-2 ligand, furfurman, did not induce TNF-α production, while stimulation with the Dectin-1 and TLR9 ligands, dZymosan and CpG, did not result in a decrease in TNF-α production in Dectin-2 KO mice (**Figure 5A**). In addition, GFP expression was not observed in either control reporter cells or Dectin-1-expressing reporter cells when stimulated with heat-killed KH2 (**Figures 5B, C**). In contrast, GFP expression in Dectin-2-expressing reporter cells was increased in a dose-dependent manner when these cells were stimulated with heat-killed KH2 (**Figure 5D**). These results suggested that Dectin-2 recognizes heat-killed KH2.

### 3.6 Effects of Dectin-1 and Dectin-2 deficiency on the promotion of skin wound healing by heat-killed KH2 administration

**Figure 6:**
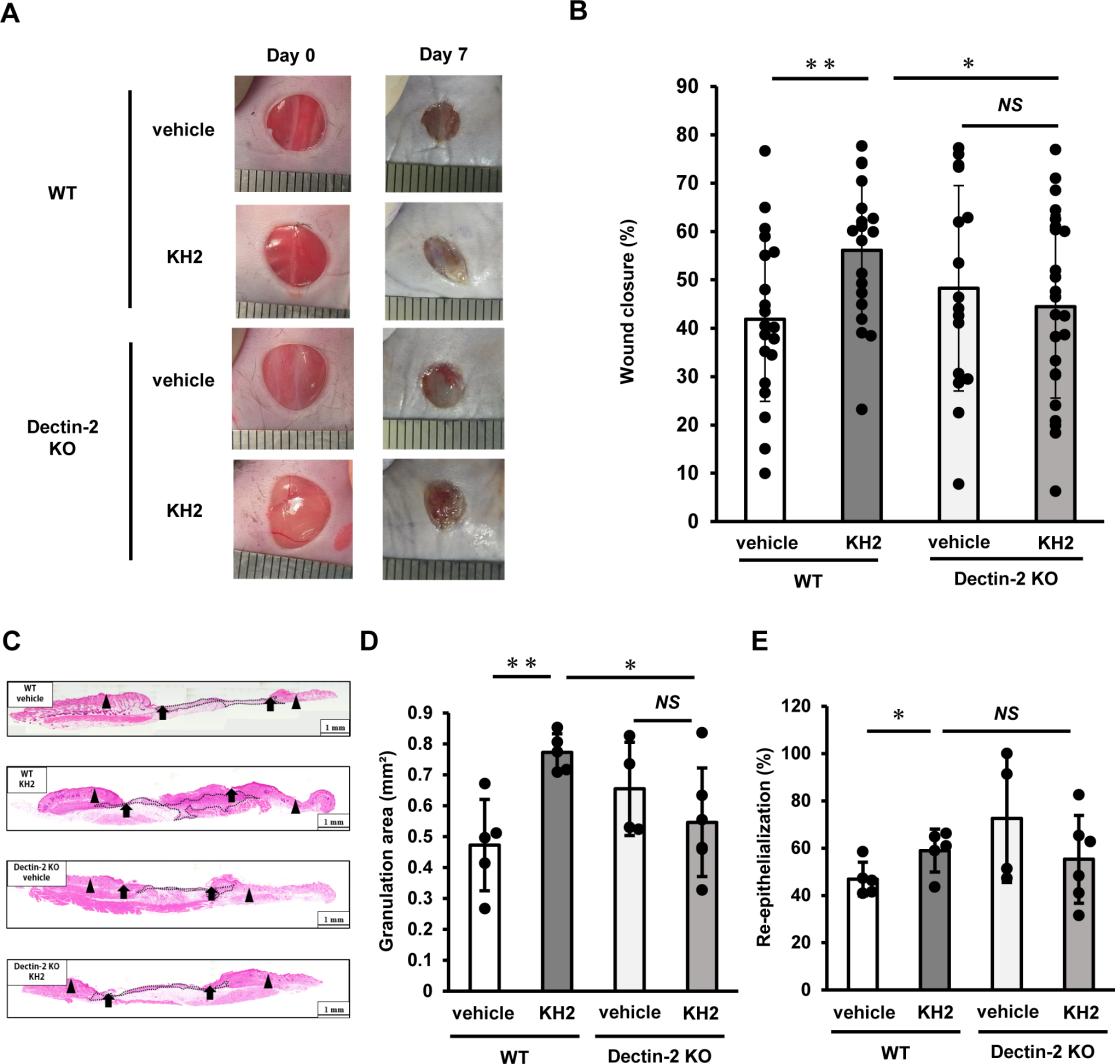
Effects of Dectin-1 and Dectin-2 deficiency on skin wound healing promoted by heat-killed KH2 administration. Wounds were created on the backs of WT mice or Dectin-2 KO mice treated with vehicle control or heat-killed KH2. Photographs **(A)** were taken, and the percentages of wound closure **(B)** were evaluated on day 7 post-wounding (n = 20–24 wounds). **(C)** Representative histological views of skin wounds on day 7. Arrowheads, arrows, and the dotted line indicate the original wound edges, granulation area, and re-epithelialized leading edges, respectively. The wound edges were defined as the border between normal epithelium and thick proliferative epithelium. **(D)** Granulation tissue area was quantified 7 days after wound creation (n = 4–6 wounds). **(E)** The re-epithelialization ratio was calculated 7 days post-wounding (n = 4–6 wounds). Each column represents the mean ± standard deviation. Results are representative of at least two independent experiments. *p < 0.05, **p < 0.01, NS, Not significant.

To further elucidate the role of Dectin-1 and Dectin-2 in promoting wound healing induced by heat-killed KH2, we investigated its involvement in wound healing using Dectin-1 KO mice and Dectin-2 KO mice. As shown in **Figures 6A, B**, WT mice treated with heat-killed KH2 showed significant acceleration of wound closure on day 7 post-wounding compared to mice treated with vehicle control. However, this effect was not observed in heat-killed KH2-treated Dectin-2 KO mice. Furthermore, similar to Dectin-2 KO mice, heat-killed KH2 did not promote wound closure in Dectin-1 KO mice (**Supplementary Figure 4**). In heat-killed KH2-treated WT mice, the granulation area was significantly increased compared to vehicle-treated mice on day 7 post-wounding, but this effect was not observed in heat-killed KH2-treated Dectin-2 KO mice (**Figures 6C, D**). The re-epithelialization rate was similar in both heat-killed KH2-treated Dectin-2 KO mice and WT mice (**Figures 6C, E**). These results suggested that Dectin-2 may be involved in promoting wound closure and granulation tissue formation, although not in heat-killed KH2-mediated re-epithelialization, and that Dectin-1, while not involved in heat-killed KH2 recognition, may contribute to promoting wound closure.

## 4 Discussion

In this study, we found that heat-killed KH2 was recognized by Dectin-2, not Dectin-1, and that Dectin-2-CARD9 signaling contributed to the acceleration of wound healing.

CARD9 is well known for its involvement in neutrophilic inflammatory responses, particularly through the production of pro-inflammatory cytokines such as TNF-α and IL-6, as well as chemokines like KC and MIP-2, which are involved in neutrophil migration. Previous studies have demonstrated that fungal infection in CARD9 KO mice leads to a marked decrease in neutrophil infiltration, accompanied by reduced production of the chemokines KC and MIP-2 (27, 28). Furthermore, in *Streptococcus pneumoniae* infection, another Gram-positive bacterium, CARD9 KO mice exhibit decreased neutrophil numbers due to reduced chemokine production (29). Consistent with these earlier studies, we observed that CARD9 KO mice had reduced neutrophil numbers and diminished production of pro-inflammatory cytokines (TNF-α, IL-6) and chemokines (KC, MIP-2) in response to heat-killed KH2 treatment compared to WT mice. These findings suggest that CARD9 is required for heat-killed KH2-induced production of inflammatory cytokines and chemokines, as well as the recruitment of neutrophils.

Neutrophils are essential for clearing pathogens from wounds. However, prolonged neutrophil infiltration can impede the resolution of inflammation and delay wound healing (30, 31). In this study, we observed a significant increase in neutrophil infiltration in heat-killed KH2-treated WT mice on day 1 post-wounding, followed by a decline by day 3, suggesting that neutrophils did not persistently accumulate at the wound site. These findings indicate that the recruited neutrophils may have contributed to accelerated wound healing. In contrast, the number of neutrophils was decreased in heat-killed KH2-treated CARD9 KO mice compared to heat-killed KH2-treated WT mice, although the kinetics of neutrophil infiltration post-wounding were similar between the two groups. While the pattern of neutrophil infiltration was comparable between heat-killed KH2-treated WT and CARD9 KO mice, the impaired wound healing observed in heat-killed KH2-treated CARD9 KO mice may indicate a potential impairment of neutrophil activity due to CARD9 deficiency. As previously mentioned, CLRs upstream of CARD9 are primarily expressed in macrophages and DCs; however, these receptors are also found in neutrophils (16) (32),. In addition, Sun et al. reported that neutrophils from CARD9-deficient mice produced less TNF-α and IL-6 in response to fungal stimulation (33). Our study showed reduced TNF-α and IL-6 production from CARD9 KO in response to heat-killed KH2, suggesting that CARD9 may be important for neutrophil function in inflammation. Additionally, our previous study demonstrated that neutrophil-derived TNF-α promotes wound healing (34). Therefore, we speculate that in this study, neutrophils producing TNF-α via the CARD9 signaling pathway may have contributed to accelerated wound healing.

Macrophages are also known to be the most important cells in skin wound healing. Macrophages are broadly classified into pro-inflammatory and anti-inflammatory macrophages (35). Both types of macrophages are important for wound healing. Pro-inflammatory macrophages accumulate early after injury and contribute to healing by phagocytosing necrotic tissue and pathogenic microorganisms, and clear apoptotic neutrophils. Subsequently, anti-inflammatory macrophages accumulate or polarize at the wound site and function to complete wound healing, through the production of growth factors (36).

In this study, the number of macrophages in heat-killed KH2-treated CARD9 KO mice was lower compared with heat-killed KH2-treated WT mice on day 1 post-wounding, with a trend toward lower numbers on day 7. The number of macrophages on day 1 post-wounding also correlated with the production of the macrophage chemokines MIP-1α and MIP-1β. Conversely, the number of macrophages on day 3 post-wounding did not correlate with chemokine production levels. In CARD9 KO mice, the discrepancy between macrophage accumulation and chemokine levels has been reported in a cryptococcal infection model, but the reason for this inconsistency was unclear (37). Investigations into expression of CCR1, -4, and -5, which are chemokine receptors for MIP-1α and MIP-1β (26), on macrophages in CARD9 KO mice will be necessary in the future.

Furthermore, in this study, we analyzed both pro-inflammatory (Ly6C+MHCII−) and anti-inflammatory (Ly6C−MHCII+) macrophages on day 7 post-wounding. We found that the proportion of pro-inflammatory macrophages was significantly lower in WT mice treated with heat-killed KH2 compared with vehicle-treated WT mice. This effect that was not observed in CARD9 KO mice.

Rodero et al. reported that a decrease in the proportion of pro-inflammatory macrophages is important for promoting healing (24). Therefore, we speculated that the decrease in pro-inflammatory macrophages also contributed to promoting healing in this study. However, regarding absolute numbers, both pro-inflammatory and anti-inflammatory macrophages were increased in heat-killed KH2-treated WT mice.

As mentioned earlier, macrophages are crucial immune cells in skin wound healing. Lucas and colleagues showed the importance of macrophages at all stages of wound healing, demonstrated by macrophage depletion at different times, indicating that depletion of both pro-inflammatory and anti-inflammatory macrophages delayed wound healing (38). Lörchner H, et al. have also shown the potential for Ly6C+MHCII− macrophages to promote healing through angiogenesis after myocardial infarction (39), suggesting that the increased levels of Ly6C+MHCII− macrophages observed in our study may have contributed to skin wound repair. Further research is needed to elucidate the role of Ly6C+MHCII− pro-inflammatory macrophages.

In the current study, we observed that heat-killed KH2 was recognized by Dectin-2, not Dectin-1. This suggests that heat-killed KH2 contains a ligand that can bind to Dectin-2. In a previous study, Yoshikawa et al. demonstrated that *L. paracasei* KW3110 strain upregulated Dectin-2 expression in macrophages, suggesting that Dectin-2 is involved in the recognition and subsequent phagocytosis of *L. paracasei* KW3110 (12). Furthermore, Bene et al. demonstrated that Dectin-2 recognizes the mucosa-binding protein of *L. reuteri* ATCC 53608 strain, leading to the induction of TNF-α and IL-6 production (40). To the best of our knowledge, while there have been some reports of Dectin-2 involvement in recognizing *Lactobacillus* spp., no studies have yet demonstrated a role for Dectin-2 in recognizing *Enterococcus* spp. Currently, the involvement of CLRs in *E. faecalis* is understood to include the role of mannose receptors (MRs) in the phagocytosis of *E. faecalis* EC-12 strain (41) and the reported involvement of Mincle in *E. faecalis* recognition (42).

Dectin-2 is a pattern recognition receptor that binds to high-mannose structures, such as α-mannan and lipoarabinomannan (18, 43). The presence of mannose in *E. faecalis* has been reported (44), suggesting that Dectin-2 may interact with the mannose components of the exopolysaccharide in heat-killed KH2. Furthermore, earlier studies have shown that MR and Dectin-2 share common ligands (45, 46). Therefore, the ligand recognized by MR may also be detected by Dectin-2. Mincle is also known to be a CLR that recognizes α-mannose (47). To investigate its potential role, we stimulated BM-DCs from Mincle KO mice with heat-killed KH2, but did not observe a decrease in TNF-α production in Mincle KO mice (**Supplementary Figure 5**). Based on these results, we conclude that Mincle is not involved in the recognition of heat-killed KH2. This result differs from a previous study that reported that Mincle recognizes *E. faecalis* (42). The previous study used *E. faecalis* that had translocated from the intestines of mice to bone marrow, whereas this study used *E. faecalis* KH2 isolated from human feces. Additionally, previous studies used LB broth for culturing, while we used MRS broth. Given that research showed strain and culture methods affect the cell wall components of LAB (44), therefore the different results could be due to differences in strain or culture methods.

Next, we assessed the potential contribution of Dectin-2 to wound healing in response to heat-killed KH2 treatment. We observed a decrease in both the percent wound closure and the granulation area in heat-killed KH2-treated Dectin-2 KO mice compared to heat-killed KH2-treated WT mice. These findings were consistent with those observed in CARD9 KO mice treated with heat-killed KH2. However, in contrast to the effects observed in CARD9 KO mice, KH2 administration did not significantly affect re-epithelialization in Dectin-2 KO mice. These results suggest that Dectin-2 recognition of KH2 is primarily involved in granulation tissue formation rather than re-epithelialization. To further elucidate the mechanisms underlying re-epithelialization, it is necessary to consider the potential involvement of other CLRs upstream of CARD9, such as Dectin-1, Dectin-3, and Mincle (46, 48). Among these, Dectin-3 has been reported to form heterodimers with Dectin-2 (49), suggesting a potential role in promoting re-epithelialization upon KH2 recognition. Since the role of Dectin-3 in KH2 recognition has not been investigated in this study, further studies are required to clarify its involvement.

Furthermore, as KH2 is a lactic acid bacterium, it likely possesses ligands for receptors other than Dectin-2, such as TLR2 and TLR9, which recognize peptidoglycan, lipoteichoic acid, and DNA (50). Both TLR2 and TLR9 have been reported to promote wound healing (51, 52) so it is possible that stimulation of these TLRs may have contributed to the observed healing effects. Therefore, further studies are needed to elucidate the roles of other receptors involved in KH2 recognition, including TLRs, in wound healing. While Dectin-1 did not seem to participate in KH2 recognition, our results indicated its potential involvement in heat-killed KH2-induced wound closure. Dectin-1 also synergizes with TLR2 to enhance cytokine production (53). Therefore, although further investigation is needed, Dectin-1 may be involved in cytokine production following heat-killed KH2 recognition by TLR2 and, consequently, in promoting wound closure.

In conclusion, this study demonstrated that topical administration of heat-killed KH2 strain induced inflammation and accelerated wound closure, granulation tissue formation, and re-epithelialization. The CARD9 pathway was shown to be involved in the wound healing-promoting effects of heat-killed KH2. In this study, we used heat-killed KH2, which has been reported to elicit an immune response equivalent to that of live bacteria, but with the advantages of reduced risk of antibiotic resistance and systemic infection (3, 54). Furthermore, as heat-killed bacteria do not proliferate, they do not cause persistent infection or inflammation. Indeed, the inflammation induced by heat-killed KH2 administration was transient in this study. These findings suggest that the topical application of heat-killed KH2 could be a promising therapeutic strategy for wound healing.

## Data Availability

The raw data supporting the conclusions of this article will be made available by the authors, without undue reservation.
