# Supplementary material for: Contribution of CARD9 signaling to wound healing in skin promoted by topical administration of heat-killed Enterococcus faecalis strain KH2 and the involvement of Dectin-2
Source: Front Immunol. 2025 Jun 12;16:1550934. doi: 10.3389/fimmu.2025.1550934 (PMC12198255; doi:10.3389/fimmu.2025.1550934)
Supplement: Supplementary file 1 [file DataSheet1.pdf]

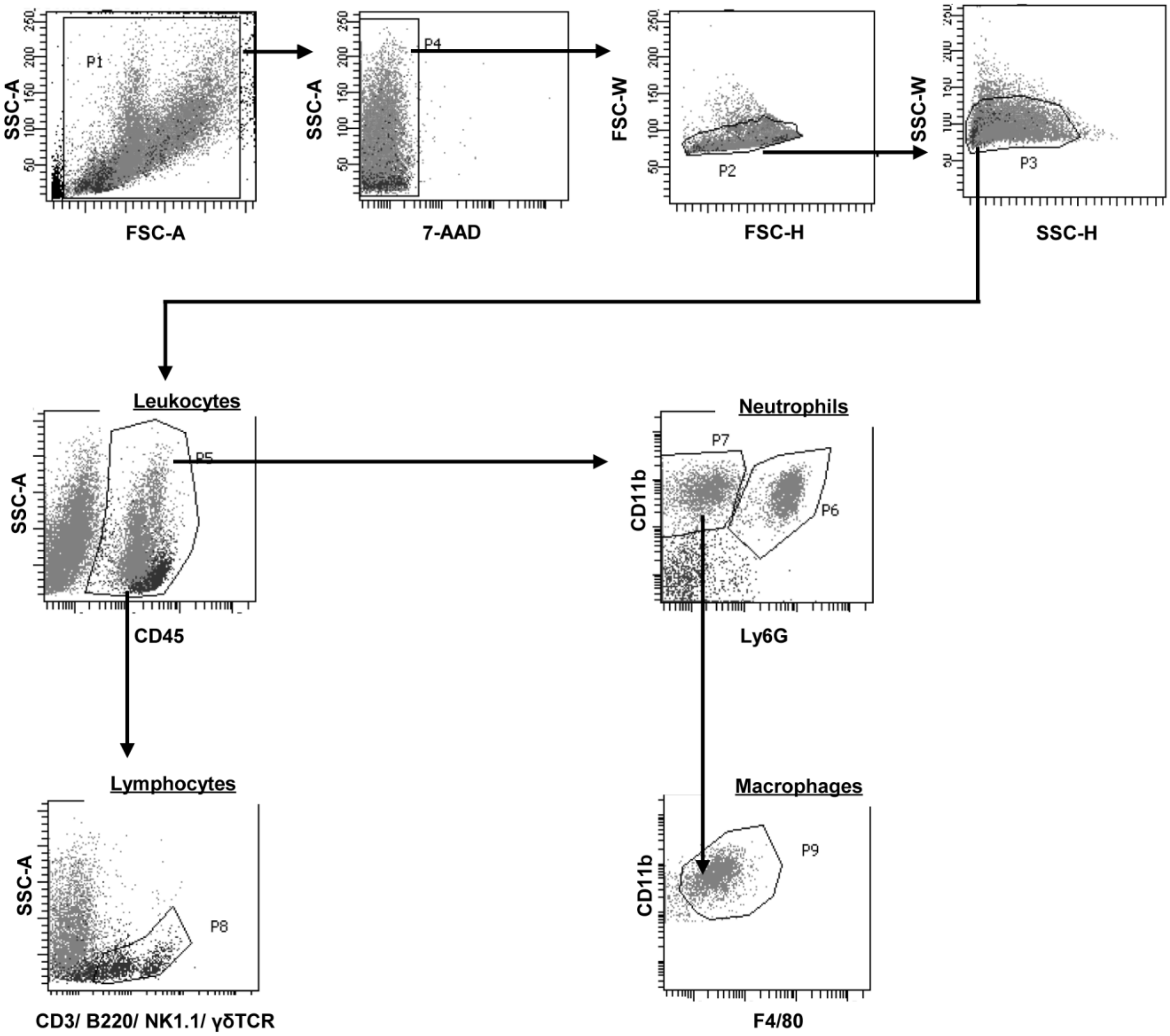

Supplemental figure 1. The gating strategy used in the analysis of the leukocyte fraction and representative scatter plots.

Cells were isolated and incubated with anti-mouse CD16/CD32 on ice for 15 min in PBS containing 1% FCS and 0.1% sodium azide. Cells were then stained with Pacific Blue-anti-CD45 mAb, APC-anti-CD11b mAb, APC/Cy7-anti-Ly6G mAb, PE-anti-F4/80 mAb, FITC-anti-CD3ε mAb, FITC-anti-NK1.1 mAb, FITC-anti-TCRγδ mAB, FITC-anti-CD45R/B220 mAb, and 7-AAD Viability staining solution. Isotype-matched irrelevant IgG was used as a control. Neutrophils and macrophages were identified as CD45+CD11b+Ly6G+cells and CD45+CD11b+F4/80 cells, respectively. Lymphocytes were identified as CD45+ cells expressing CD3, NK1.1, TCRγδ, or B220. Stained cells were analyzed using a BD FACS Cant II flow cytometer.

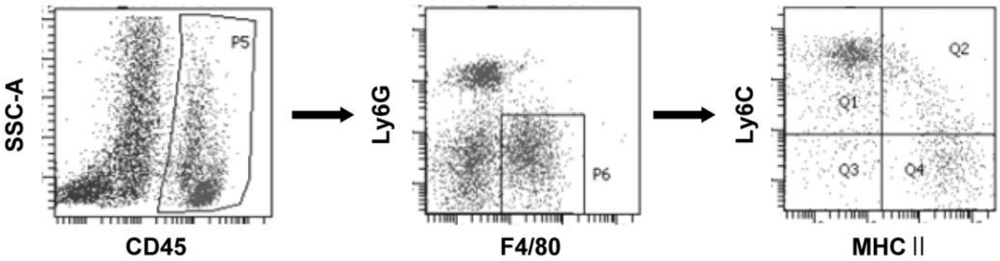

Supplemental figure 2. The gating strategy used in the analysis of the macrophage phenotype.

Cells were isolated and incubated with anti-mouse CD16/CD32 on ice for 15 min in PBS containing 1% FCS and 0.1% sodium azide. Cells were then stained with Pacific Blue-anti-CD45 mAb, APC/Cy7-anti-Ly6G mAb, PE-anti-F4/80 mAb, APC-anti-Ly6C mAb, FITC-IA/IE (MHC II) mAb, and 7-AAD Viability staining solution. After excluding doublet cells and dead cells as described in Supplemental Figure 1, we gated on CD45+F4/80+Ly6G- cells and analyzed the expression of Ly6C and MHCII. Isotype-matched irrelevant IgG was used as a control. Pro-inflammatory and anti-inflammatory macrophages were identified as CD45+F4/80+Ly6G-Ly6C+MHCII- cells and CD45+F4/80+Ly6G-Ly6C-MHCII+ cells, respectively. Stained cells were analyzed using a BD FACS Cant II flow cytometer.

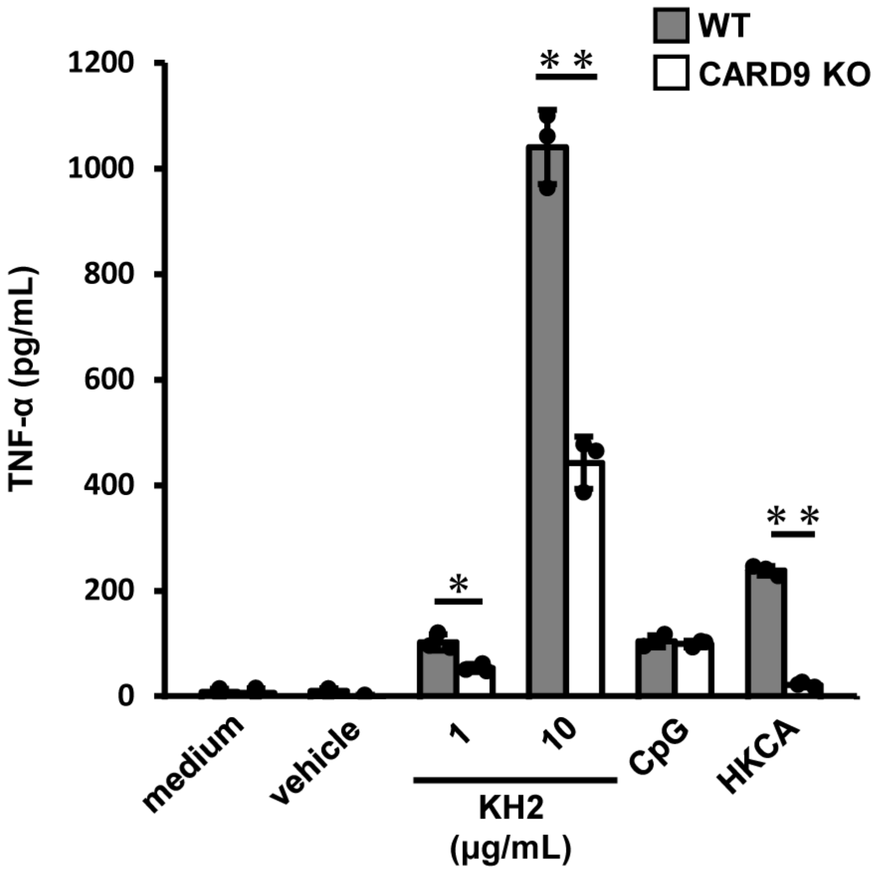

Supplemental figure 3. Effect of CARD9 deficiency on TNF-α production in BM-DCs stimulated by heat-killed KH2.

BM-DCs were prepared from WT mice and CARD9 KO mice and stimulated with heat-killed KH2 (1, 10 µg/mL), CpG (1 µg/mL), and HKCA (MOI 3) for 24 h. Production of TNF-α in the culture supernatants was analyzed (n = 3). Each column represents the mean ± standard deviation. Results are representative of at least two independent experiments. ＊$p < 0.05$, ＊＊ $p < 0.01$.

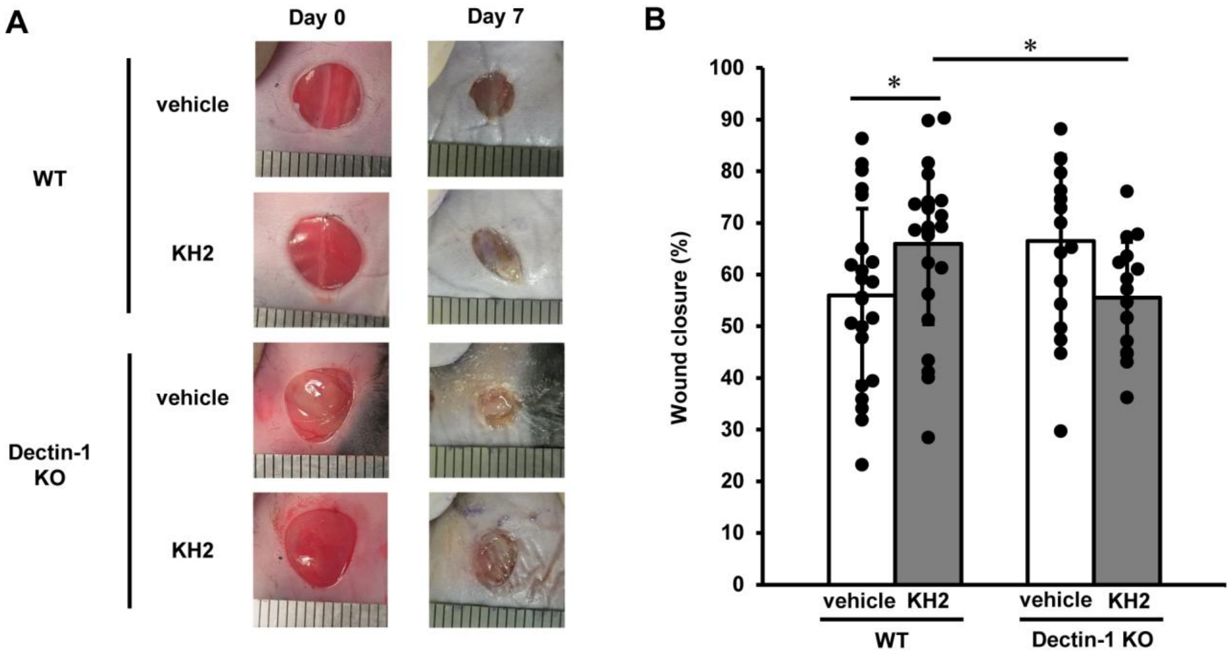

Supplemental figure 4. Effects of Dectin-1 deficiency on the promotion of wound closure y heat-killed KH2 administration.

Wounds were created on the backs of WT mice or Dectin-1 KO mice treated with vehicle control or heat-killed KH2. Photographs (A) were taken, and the percentages of wound closure (B) was evaluated on day 7 post-wounding (n = 16–24 wounds). Each column represents the mean ± standard deviation. Results are representative of at least two independent experiments. $*p < 0.05$.

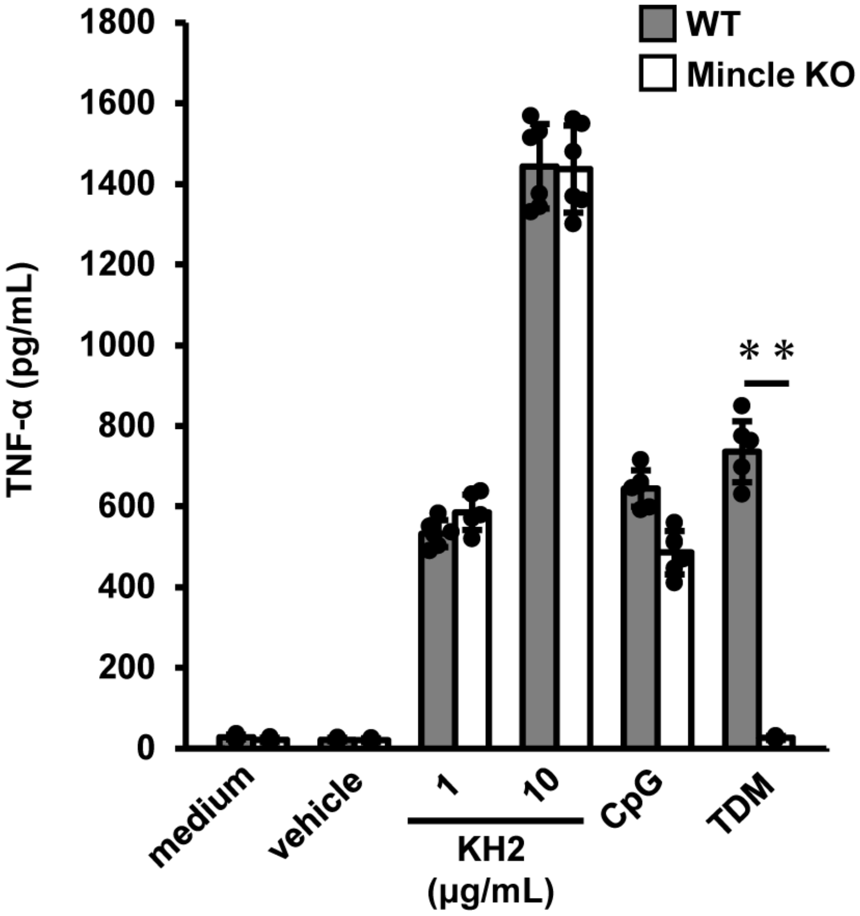

Supplemental figure 5. Effect of Mincle deficiency on TNF-α production in BM-DCs stimulated by heat-killed KH2.

BM-DCs were prepared from WT and Mincle KO mice and stimulated with heat-killed KH2 (1, 10 μg/mL), CpG (1 μg/mL), or TDM (5 μg/mL) for 24 h. Production of TNF-α in the culture supernatants was analyzed (n = 6). Each column represents the mean ± standard deviation. Results are representative of at least two independent experiments.
＊＊ $p < 0.01$.
